# Methodological Issues on the Importance of Instrument Validation in Cross-Sectional Health Research

**DOI:** 10.29252/beat-080109

**Published:** 2020-01

**Authors:** Mehrdad Amir-Behghadami, Masoumeh Gholizadeh, Ali Janati

**Affiliations:** 1 *Iranian Center of Excellence in Health Management (IceHM), Department of Health Service Management, School of Management and Medical Informatics, Tabriz University of Medical Sciences, Tabriz, Iran*; 2 *Student Research Committee (SRC), Tabriz University of Medical Sciences, Tabriz, Iran*

***Dear Editor,***

Recently, we read with great interest the article authored by Jadidi *et al.* [1] that was entitled “Is emergency medical services (EMS) in Islamic Republic of Iran practical and efficient in facing Ebola?” and published in Bull Emerg Trauma in 2019, in 7^th^ volume and 3^rd^ issue. First of all, we would like to extend our gratitude to the authors of this article. Although the mentioned study was appropriate and valuable, there was a fundamental flaw in the method, which has led to an ambiguous interpretation of the findings. Therefore, the purpose of this letter is to raise concerns about the data collection instrument and emphasize the importance of reporting its validity and reliability in cross-sectional studies. 

In the mentioned study, the authors evaluated the efficacy and preparedness of Emergency Medical Services (EMS) in Islamic Republic of Iran to face Ebola [1]. In this regard, either a new instrument is designed or an existing instrument is used [2, 3]. If they had used an existing instrument, they would have to be culturally adapted before being used in the Iranian context. However, it is unclear whether they have developed a new instrument for their evaluations or have used the available instrument, and it is necessary to report its validity and reliability indicators.

Guidelines like the Strengthening the Reporting of Observational Studies in Epidemiology (STROBE) [4] or STROBE-ME [5] have been developed to guide researchers on criteria that may assist in conducting their own research. Hence, in order to be more clear, it is recommended that researchers to conduct their studies in accordance with the STROBE statement, as its use decreases the risk of flawed reporting and increases the quality.

## Conflict of Interest:

 Not to declare.

## References

[1] Jadidi A, Irannejad B, Bahrami P, Moradi Y, Zaker Tarzam MR (2019). Is Emergency Medical Services (EMS) in Islamic Republic of Iran Practical and Efficient in Facing Ebola?. Bull Emerg Trauma..

[2] Amir-Behghadami M, Janati A (2019). A Closer Look at the Validity and Reliability of the Persian Versions of National Institute of Health Stroke Scale and Modified National Institute of Health Stroke Scale in Hospitalized Patients. Galen Medical Journal..

[3] Amir Behghadami M, Janati A, Sadeghi-Bazargani H, Gholizadeh M, Rahmani F, Arab-Zozani M (2019). Developing and validating an instrument to assess non-hospital health centers' preparedness to provide initial emergency care: a study protocol. BMJ Open..

[4] von Elm E, Altman DG, Egger M, Pocock SJ, Gøtzsche PC, Vandenbroucke JP (2007). STROBE Initiative The Strengthening the Reporting of Observational Studies in Epidemiology (STROBE) statement: guidelines for reporting observational studies. AnnIntern Med.

[5] Gallo V, Egger M, McCormack V, Farmer PB, Ioannidis JP, Kirsch-Volders M (2011). STrengthening the Reporting of OBservational studies in Epidemiology--Molecular Epidemiology (STROBE-ME): an extension of the STROBE Statement. PLoS Med.

